# The awareness of behavioral addictions in general practitioners- An epidemiological report

**DOI:** 10.1192/j.eurpsy.2024.858

**Published:** 2024-08-27

**Authors:** O. Vasiliu

**Affiliations:** Psychiatry, Dr. Carol Davila University Emergency Central Military Hospital, Bucharest, Romania

## Abstract

**Introduction:**

Although the research on behavioral addictions (BAs) is continuously developing, the awareness about this category of disorders and their important negative consequences still remains a problem for many physicians. This phenomenon is associated with delayed diagnosis and treatment initiation, lack of valid epidemiological data about these pathologies, and overall lower quality of life in these patients.

**Objectives:**

The main objective of this study was to explore the awareness of GPs on the general diagnosis criteria of BAs.

**Methods:**

An online questionnaire addressed to general practitioners (GPs) investigated the level of their knowledge regarding the main criteria for diagnosis in five more commonly reported BAs, i.e., gambling disorder, problematic Internet use, cell phone addiction, food addiction, and shopping addiction. The questionnaire included 50 items and required 20-25 minutes to complete. The answers were anonymized.

**Results:**

Answers from 12 GPs were analyzed, with an 80% completion rate. Gambling disorder was the only diagnosis recognized by all the respondents, followed by shopping addiction (50%) and abusive Internet use (33.3%). Lack of time to screen for these disorders was the most frequently invoked reason for not including instruments dedicated to BAs in the regular visits to the GPs. The Internet was admitted by all the respondents as their source of information about BAs.

**Conclusions:**

There is an acute need to improve the knowledge of GPs about the existence and consequences of BAs in order to increase the probability of early detection and treatment initiation for these patients. It is expected that Internet-based campaigns for increasing GPs will benefit BAs patients in the long term.

**Disclosure of Interest:**

None Declared

